# The role of butyrylcholinesterase in the regulation of cognitive dysfunction in minimal hepatic encephalopathy: A potential blood marker of disease evolution

**DOI:** 10.3389/fneur.2022.900997

**Published:** 2022-10-20

**Authors:** Xuhong Yang, Pei Dang, Wenxiao Liu, Wanlong Ma, Xin Ge, Kai Zhu, Minglei Wang, Xueying Huang, Xiangchun Ding, Xiaodong Wang

**Affiliations:** ^1^School of Clinical Medicine, Ningxia Medical University, Yinchuan, China; ^2^Department of Radiology, General Hospital of Ningxia Medical University, Yinchuan, China; ^3^Department of Infectious Diseases, General Hospital of Ningxia Medical University, Yinchuan, China

**Keywords:** minimal hepatic encephalopathy, cognition function, regional homogeneity, butyrylcholinesterase, acetylcholine

## Abstract

**Background and aims:**

Patients with cirrhosis commonly experience minimal hepatic encephalopathy (MHE), and alterations in neurotransmitters have been thought to be related to cognitive function. However, the relationship between alterations in peripheral and central butyrylcholinesterase (BuChE) with MHE disease progression remains unknown. As such, this study was designed to investigate potential changes in peripheral and central BuChE activity and their effects on cognitive function in the context of MHE.

**Materials and methods:**

We enrolled 43 patients with cirrhosis secondary to hepatitis B, 20 without MHE and 23 with MHE, and 25 with healthy controls (HC). All the selected subjects underwent resting-state functional MRI, and the original images were processed to obtain the regional homogeneity (ReHo) brain maps. Thereafter, the correlation of BuChE activity with ReHo, number connection test of type A (NCT-A), and digital symbol test (DST) scores with MHE patients were analyzed using *Person* correlation analysis. Meanwhile, we purchased 12 Sprague-Dawley (SD) rats and divided them into an experimental group (*n* = 6) and a control group (*n* = 6). The rats in the experimental group were intraperitoneally injected with thioacetamide (TAA) to prepare MHE model rats. After modeling, we used the Morris water maze (MWM) and elevated plus maze (EPM) to assess the cognition function and exploratory behavior of all rats. The activity of serum, hippocampus, and frontal lobe tissue BuChE was detected by ELISA.

**Results:**

BuChE activity gradually decreased among the HC, patients with cirrhosis, and MHE groups (all *P* < 0.01). We observed a linear correlation between serum BuChE and NCT-A and DST scores in MHE patients (all *P* < 0.01). We noted that BuChE activity can negatively correlate with ReHo values in the left middle temporal gyrus and left inferior temporal gyrus, and positively correlate with ReHo values in the right inferior frontal gyrus, and also found that the peripheral BuChE activity of MHE rats was significantly lower than their control counterparts, and the BuChE activity in frontal lobe extracts was significantly higher than the control rats (all *P* < 0.05).

**Conclusion:**

The altered activity of BuChE may contribute to cognitive impairment in MHE patients, which may be a potential biomarker of disease evolution in the context of MHE.

## Introduction

Approximately two million deaths each year are caused by liver disease, of which one million are caused by cirrhosis and its complications at a global scale (1). Hepatic encephalopathy (HE) is a serious complication of liver failure, which is a complex neuropsychiatric syndrome caused by damage to the central nervous system (2, 3). Minimal hepatic encephalopathy (MHE) neurocognitive changes are an important feature in the early stage of HE. The patient has no obvious disturbance of consciousness, but only manifests as a decline in cognitive judgment ability, attention, and memory (4, 5), with atypical clinical signs or symptoms, but cognitive impairment can be detected by appropriate neuropsychological testing (6). The occurrence and development of MHE will significantly reduce the quality of life of patients (7), and also cause transient mild cognitive impairment, increasing the risk of various accidents and occupational disabilities. Therefore, early diagnosis and treatment are urgently needed to improve the prognosis of patients.

At present, the pathogenesis of MHE is still unclear, and it is still based on the ammonia poisoning theory (8). With in-depth research, the defects of the brain neurotransmitter system are considered to be related to the occurrence of MHE, especially the glutamate and monoaminergic mechanisms, γ-aminobutyric acid, and serotonergic systems have been reported in animal models of liver disease and patients with liver disease (9–11). At the same time, some studies have also described the effect of the cholinergic system on MHE cognitive function (12), but these studies focused on acetylcholinesterase (AChE); thus far, there have been few studies on alterations in the BuChE during liver failure, with no meaningful conclusions drawn yet. But in-depth investigation and research of BuChE, a more important function of BuChE have been proposed, including memory, selective attention, and behavioral response (13, 14). Petrov et al. (15) and others found that BuChE can be expressed in specific brain areas, such as the amygdala, hippocampus, and frontal lobe, seems to be involved in the development of certain aspects of the nervous system, and expresses nicotinic acetylcholine (ACh) receptors. This suggests that BuChE may regulate the functional state of cells by hydrolyzing the neurotransmitter ACh.

Resting-state functional magnetic resonance imaging (fMRI) is a non-invasive imaging method, which uses hemoglobin as the endogenous contrast agent, and it is a non-invasive technology to image the increase of oxyhemoglobin and the decrease of deoxyhemoglobin caused by the rapid changes in neuronal activity (16). It is the main imaging technology for the study of brain cognitive functions. At present, there are many analytical methods for fMRI, among which regional homogeneity (ReHo) analysis can effectively map the regional activity level of the brain (17) and reflect the consistency of the local neuronal activity level (18). The increased ReHo represents the increased consistency of the local neuronal activity and vice versa. A number of previous studies have investigated the influence of blood ammonia on brain neuronal activity by analyzing the correlation between blood ammonia and brain area low-frequency fluctuation (ALFF) (19).

The BuChE plays a distinct and important role in the regulation of cognitive function; according to prior research (20, 21), it is possible to provide insight into the pathophysiological basis for impaired cognition in people experiencing MHE by studying the changes in this enzyme. Accordingly, this study aimed to explore the underlying changes in peripheral and central BuChE activity in the context of MHE and their effects on cognitive function and determine whether BuChE is correlated with clinical parameters and reflects the stage of the disease at the cognitive (NCT-A, DST score).

## Materials and methods

### Subjects

This prospective study was approved by the Medical Research Ethics Committee of the local hospital (KYLL-2021-841). A total of 43 patients were admitted to the Department of Infectious Diseases of the General Hospital of Ningxia Medical University from April 2020 to June 2021 and diagnosed with post-hepatitis B cirrhosis. In addition, 25 healthy volunteers matched for gender, age, and education level (HC group) were recruited during the same period. Exclusion criteria included: (1) previous history of other neuropsychiatric disorders or psychotropic substance abuse; (2) history of parenchymal brain disease (brain tumor, stroke, traumatic brain injury, and metabolic brain diseases) as assessed by medical history and routine imaging; (3) previous history of alcohol abuse or alcoholic cirrhosis; (4) suffering from diabetes, hypertension, or other chronic metabolic diseases; (5) inability to complete the number connection test-A (NCT-A) and digital symbol test (DST) scales due to visual acuity, lack of education, and so on; and (6) contraindications to MRI, such as the presence of metal implants in the body. MHE was diagnosed by two experienced radiologists, and each patient underwent two neuropsychological assessments (NCT-A and DST) and an fMRI examination. Those who had positive results on both NCT-A and DST were considered to be in the MHE group according to the final report of the working party at the 11th World Congress of Gastroenterology (22), and only one positive result was included in the group of simple liver cirrhosis. In this study, informed consent was obtained for experimentation with all subjects.

A total of 12 SD rats (6–8 weeks, 200–260 g) were purchased from the Experimental Animal Center of Ningxia Medical University. The animals were randomly divided into an experimental group (*n* = 6) and a control group (*n* = 6) and fed with ordinary feed (Jiangsu, Xietong Bio-engineering, China) (23). We kept rats for 1 week without experimentation to allow them to adjust to their new environment, a standard laboratory environment was provided for them (with a temperature of 20–22°C, relative humidity of 65–70%, and a cycle of 12 h of light and darkness). Model preparation: The MHE model was prepared by intraperitoneal injection of thioacetamide (TAA) (Macklin Biochemical, China), while the rats in the control group were intraperitoneally injected with normal saline. The initial dose was 150 mg/kg, 3 times a week, for a total of 12 weeks. Referring to the method of Li et al. (24), the drug dose was adjusted appropriately according to the increase or decrease in body weight. MHE rats' inclusion criteria were from Ding et al. (25). This study was approved by the Animal Research Committee of Ningxia Medical University (IACUC-NYLAC-2021-114).

### MR data acquisitions

MR images were acquired using a GE Architect 3.0T MR scanner and a 48-channel magnetically sensitive coil was used. The subjects were asked to remain as quiet as possible before the scan, and the head was immobilized with a sponge to reduce the head movement. All the patients underwent conventional MRI scanning and resting-state BOLD-fMRI. The conventional MRI scans included T1FLAIR (TR = 2,000 ms, TE = 20 ms) and T2WI (TR = 4,000 ms, TE = 107 ms). Thereafter, resting-state BOLD-fMRI was performed using Ax BOLD rest 36sl sequence, TR = 2,000, TE = 30, flip angle = 90°, FOV = 250 × 250 mm, matrix = 64 × 64, number of layers = 35, slice thickness = 3.6 mm, scan dynamics = 180 times, and scan time = 6 min.

### Image preprocessing

The acquired raw images were then preprocessed in MATLAB2012a using the Resting State Brain Imaging Data Processing and Analysis Tool (DPARSF_V4.5, http://www.restfmri.net) using the following steps: (1) The data from the first 10 time points were excluded to exclude the effects on the results due to non-homogeneous magnetic field at the beginning of the scan and the subject's non-adaptation; (2) time correction: the resting images of each individual were time-corrected using the central level of the scan as the reference layer; (3) head movement correction: the head movement parameters of the MRI scan were also calculated, including the translation and rotation parameters in the x, y, and z directions, and images with excessive head movement (translation >2.0 mm or rotation of 2.0°in the head movement parameters) were excluded; (4) The corrected images were then normalized to a standard MNI (Montreal Neurological Institute) template and each voxel was resampled into 3 × 3 × 3mm^3^ voxels; (5) The normalized data were thereafter subjected to de-linearization drift and band-pass filtering (0.01–0.08 Hz) to reduce the potential drift at low frequencies and the high frequency effects of respiration as well as the heartbeat and also to remove the effects of covariates, including cerebral white matter signal and the cerebrospinal fluid signal.

#### ReHo analysis

ReHo images use Kendall's coefficient concordance (also known as ReHo values) to measure the similarity of a voxel to several of its surrounding voxels, thus indirectly reflecting the synchrony of the neuronal activity in the brain (26). The calculation of the ReHo parameter was based on the synchronization of each voxel with its neighboring 26 voxels in the time sequence to obtain an optimal ReHo map for each subject.

### Serum BuChE quantification

Patients fasted overnight (for ~12 h) before blood collection and were in the decubitus position during the sampling. The specimens were placed in an ice bath immediately after collection and the serum was immediately separated after centrifugation and tested by ion exchange. The serum BuChE activity was measured by an OLYMPUS AU5400 analyzer (Beckman Coulter, Brea, CA, USA).

### Morris water maze to detect spatial memory

The Morris water maze (MWM) was 200 cm in diameter and 55 cm in height, divided into four quadrants, with a water level of 37 cm and a temperature of 22 ± 2°C. The platform used corresponds to a cylinder with a diameter of 12 cm and a height of 35 cm, of which 2 cm is below the water's surface. The MWM was surrounded by a canvas to avoid the interference of sunlight in the experiment. The walls of the water maze are pasted with plastic plates of different shapes in four different directions (east, west, south, and north) to allow animals to identify their location. A computerized video tracking system (SMART 3.0, Panlab, Spain) was used to record behavioral data. The test lasted for 6 days, and the rat was trained four times per day at regular intervals, from four designated starting points (east, south, west, and north) facing the wall of the barrel, and the average of the four training latencies was used as the daily learning performance of the rat. Each trial ended with the animal finding the platform or 60 s, after which, if the animal did not reach the hidden platform, it was placed on the platform for 10 s. On the 6th day of the experiment, the platform was removed and the rat was placed in the water at any of the same entry points, and the swimming trajectory of rats within 60 s, the residence time in the target quadrant, and the number of times crossing the target quadrant were recorded.

### Elevated plus maze to detect autonomous activity and exploratory behavior

The elevated plus maze (EPM) is composed of two open arms and two closed arms, which are crossed vertically with each other (arm width: 10 cm, arm length: 60 cm, height of wall: 40 cm). At the beginning of the experiment, the rat was placed in the central area with its head facing the open arm, and the experiment time was 10 min. The behavior of rats in the EPM was recorded using a computerized video tracking system (SMART 3.0, Panlab, Spain). During the test session, the time and the number of times it enters open and closed arms were measured automatically by SMART 3.0. The ratio of entries into the open arms (the number of times the rats entered the open arms/the total number of times the rats entered both arms) and the ratio of time spent in the open arms (time spent in open arms/total time spent in both arms) were calculated manually.

### Enzyme-linked immune-sorbent assay

BuChE activity was measured with an ELISA kit (A025-1, Nanjing Jiancheng, China). Rats were euthanized and the hippocampal frontal lobe tissue was dissected in cold conditions (4°C) and weighed. The brain tissues were mechanically homogenized in 0.9% normal saline, according to a 1:9 ratio of weight (mg):volume (μl), under ice-water bath conditions. The homogenate was then centrifuged at 3,000 r/min for 10 min, and the supernatant was extracted for concentration determination. The experimental steps were performed according to the instructions of the ELISA kit and the total protein concentration in the hippocampal homogenate of rats in each group was normalized (BCA). The serum was drawn off and stored at −80°C for BuChE activity estimation.

### Statistical analysis

GraphPad Prism software version 9.3 (GraphPad Software, USA) was used to plot graphs and comparison. Statistical analyses were performed by using SPSS 23.0. Categorical data (sex) were expressed by (%), and the chi-square test was used to compare the differences between groups. Shapiro–Wilk test was used to verify the normality of all the measurement data. For the measurement data with a normal date, the student's *t*-test was used to compare the differences between the two groups, and ANOVA was used to compare the differences among the three groups, which were presented as mean with standard deviation. For the measurement data of non-normal date, the Mann-Whitney *U*-test was used to compare the differences between the groups, which were presented as the medians with the accompanying interquartile ranges.

#### Correlation analysis

This analysis was conducted to investigate the potential association between the venous BuChE activity and ReHo values in patients with MHE by using the Resting-State fMRI Data Processing Toolkit (DPARSF_V4.5, http://www.restfmri.net) and xjview (27). The correlations between BuChE activity and mean ReHo values of brain regions in MHE patients were determined using the *Person* correlation analysis and the brain regions where correlations existed were analyzed. The statistical threshold was set at *P* < 0.05 (after AlphaSim correction).

## Results

### Demographic and clinical information

The detailed demographic characteristics and clinical data for all participants are summarized in Table 1. There were no significant differences observed in regard to gender, age, or education level among the three groups (Table 1). However, compared with the HC and liver cirrhosis groups, the DST score in the MHE group decreased, and the NCT-A score increased (all *P* < 0.05). There was no significant difference in NCT-A and DST scores between the HC and cirrhosis groups (*P* > 0.05) (Table 1).

**Table 1:** Demographic and physiologic data of the studied cohort.

	**HC group (*n* = 25)**	**Cirrhosis group (*n* = 20)**	**MHE group (*n* = 23)**	***χ^2^/F/z/t* value**	***P-*** **value**
Sex (% of male patients)	56.0%	55.0%	60.9%	0.180	0.914^a^
Age (years)	46.9 ± 9.4	48.3 ± 10.6	48.1 ± 9.8	1.881	0.161^b^
Years of education	8.0 ± 1.2	7.7 ± 1.6	7.6 ± 1.4	2.212	0.118^b^
NCT-A (seconds)	34.39 (30.74, 41.64)	37.09 (31.98, 42.51)	68.79 (59.60, 90.83)	−0.411	0.681^†^^#^
				−5.521	<0.001^‡^^#^
				−4.626	<0.001^§^^#^
DST (score)	46.6 ± 8.3	43.4 ± 3.3	21.7 ± 7.2	−1.620	0.113^†^^&^
				10.953	<0.001^‡^^&^
				12.614	<0.001^§^^&^

DST, digital symbol test; NCT-A, number connection test of type A; BuChE, butyrylcholinesterase; HC, healthy control; MHE, minimal hepatic encephalopathy.

a χ^2^ test of three groups.

b One-way analysis of variance test among three groups.

† HC and cirrhosis groups.

‡ HC and MHE groups.

§ Cirrhosis and MHE groups.

# Mann–Whitney *U*-test.

& Student's *t*-test.

### Changes in serum BuChE activity with three groups

**Figure 1:**
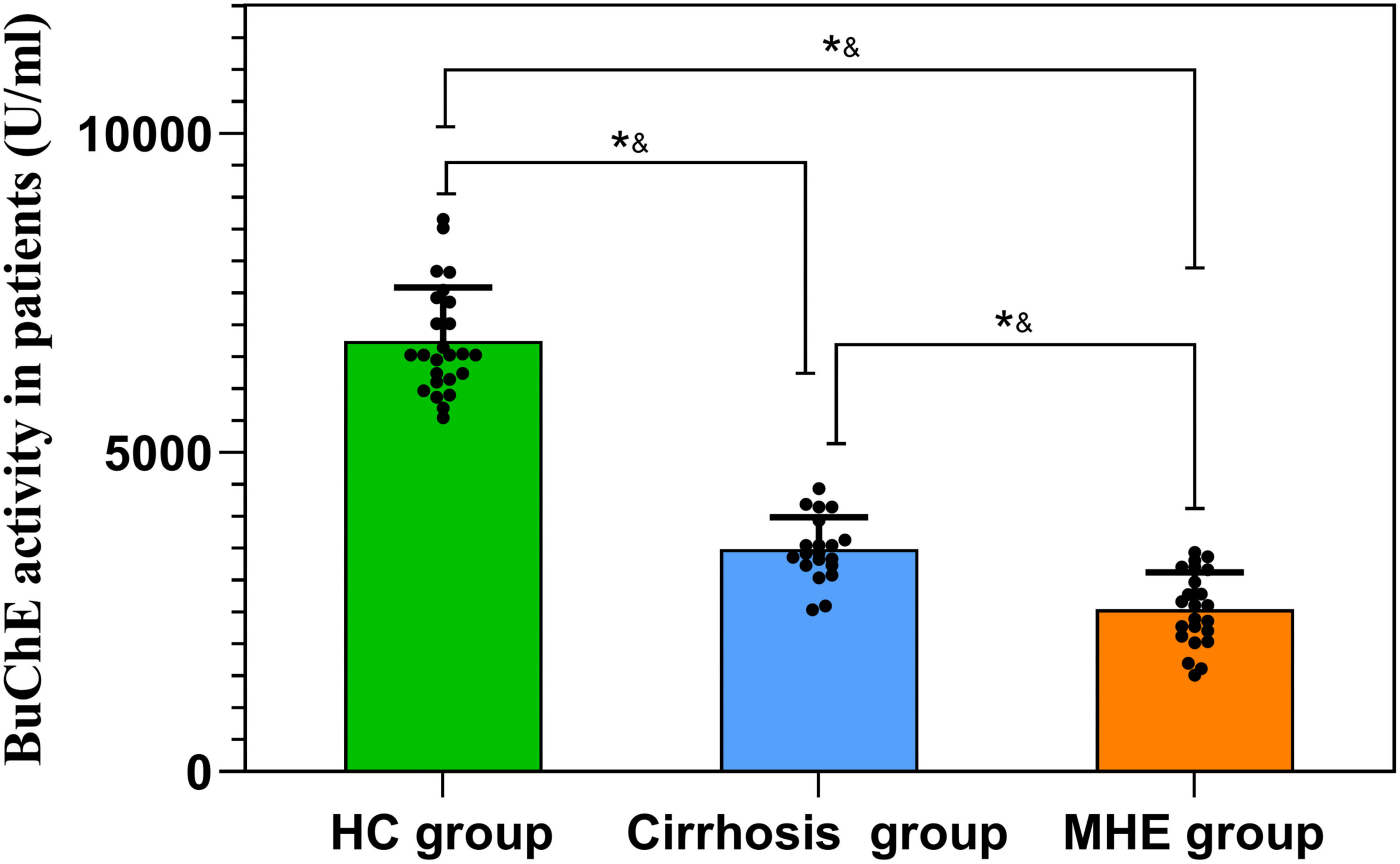
Changes in serum BuChE activity with three groups, *P < 0.05, ^&^Student's *t*-test.

We investigated the effect of MHE on the BuChE activity in the serum. Our results reveal an alteration of the BuChE activity in the serum of MHE patients. The BuChE activity gradually decreased between the three groups (all *P* < 0.05) (Figure 1).

### Correlation analysis of BuChE activity with NCT-A and DST scores

**Figure 2:**
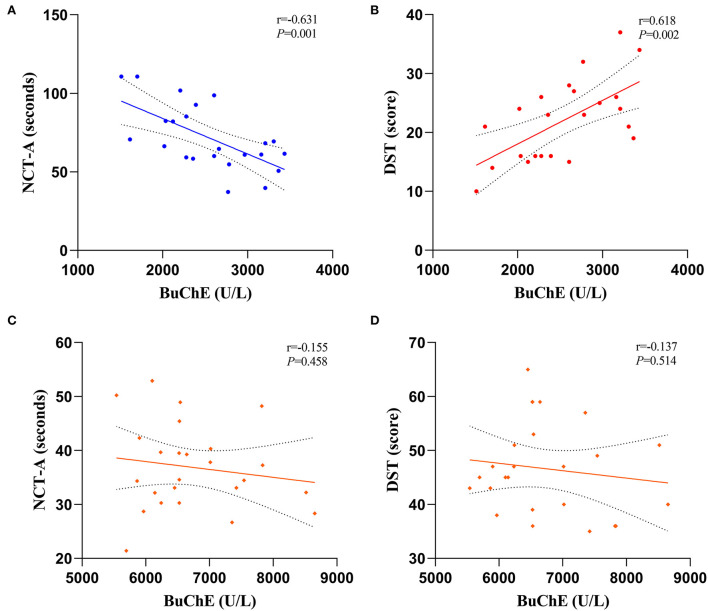
Correlations between BuChE activity with NCT-A and DST scale scores, **(A,B)** are MHE groups and **(C,D)** are HC groups.

Figure 2 shows the correlation between BuChE with NCT-A and DST scores in MHE and HC groups. BuChE activity in the MHE group exhibited a significant negative correlation with NCT-A score (*r* = −0.631, *P* = 0.001) (Figure 2A) but a significant positive correlation with DST score (*r* = 0.618, *P* = 0.002) (Figure 2B). However, BuChE activity in the HC group did not show any significant correlation with NCT-A and DST scores (*r* = −0.155, *P* = 0.458) (Figure 2C); (*r* = −0.137, *P* = 0.514) (Figure 2D).

### Correlation analysis of BuChE activity with ReHo value

**Figure 3:**
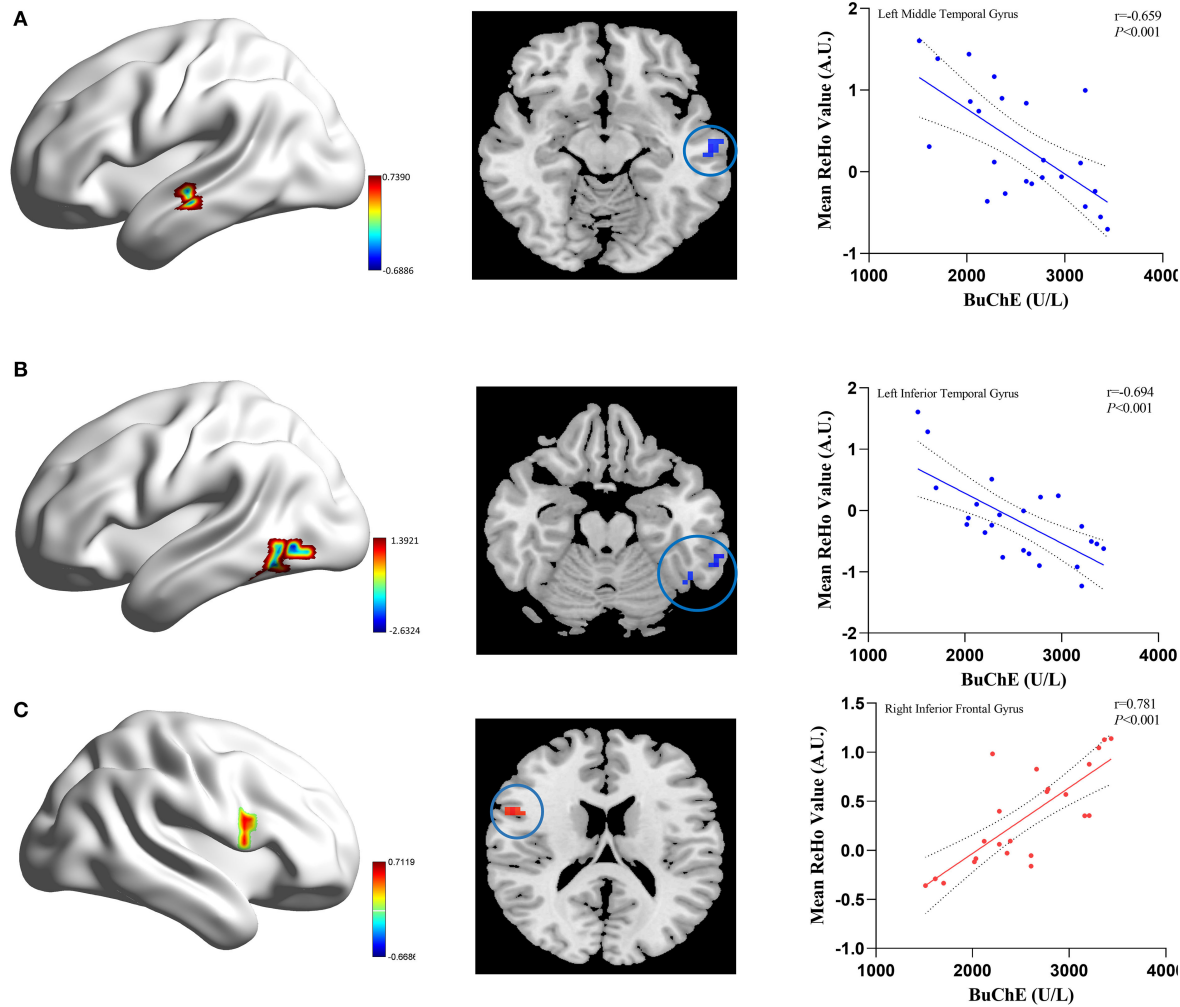
The correlation between the mean ReHo values and BuChE activity in the MHE patients. The BuChE activity negatively correlates with ReHo values in the left middle temporal gyrus **(A)**, left inferior temporal gyrus **(B)** (blue color), and positively correlate with ReHo values in the right inferior frontal gyrus **(C)** (red color).

Figure 3 shows the correlation between ReHo and BuChE activity in the MHE group. In patients with MHE, the serum BuChE activity displayed a negative correlation with ReHo in the left middle temporal gyrus and left inferior temporal gyrus, and a positive correlation with ReHo in the right inferior frontal gyrus (*P* < 0.05, after AlphaSim correction).

### Spatial memory performance assays

In the positioning navigation experiment, with the increase of training times, the latency (time to reach the original platform) of the control rats gradually decreased than their MHE counterparts, and there were differences between the two groups on the fourth and fifth days (*P* < 0.05) (Table 2). The above results indicated that the learning ability of the MHE rats was damaged compared to the control rats.

**Table 2:** MWM results of control and MHE groups.

**Groups**	**Control group** **(*n* = 6)**	**MHE group** **(*n* = 6)**	***t*** **-value**	***P*** **-value**
Day 1 (s)	43.5 ± 12.8	46.4 ± 16.1	−0.369	0.718^&^
Day 2 (s)	30.5 ± 10.4	33.5 ± 22.6	−0.302	0.768^&^
Day 3 (s)	27.7 ± 8.2	34.3 ± 15.9	−0.930	0.369^&^
Day 4 (s)	14.4 ± 4.3	30.4 ± 10.1	−3.657	0.003^&^
Day 5 (s)	11.6 ± 6.1	26.6 ± 11.8	−2.828	0.014^&^

All values are shown as mean ± standard deviation ($\bar{\text{x}}$ ± *s*);

& Student's *t*-test.

**Figure 4:**
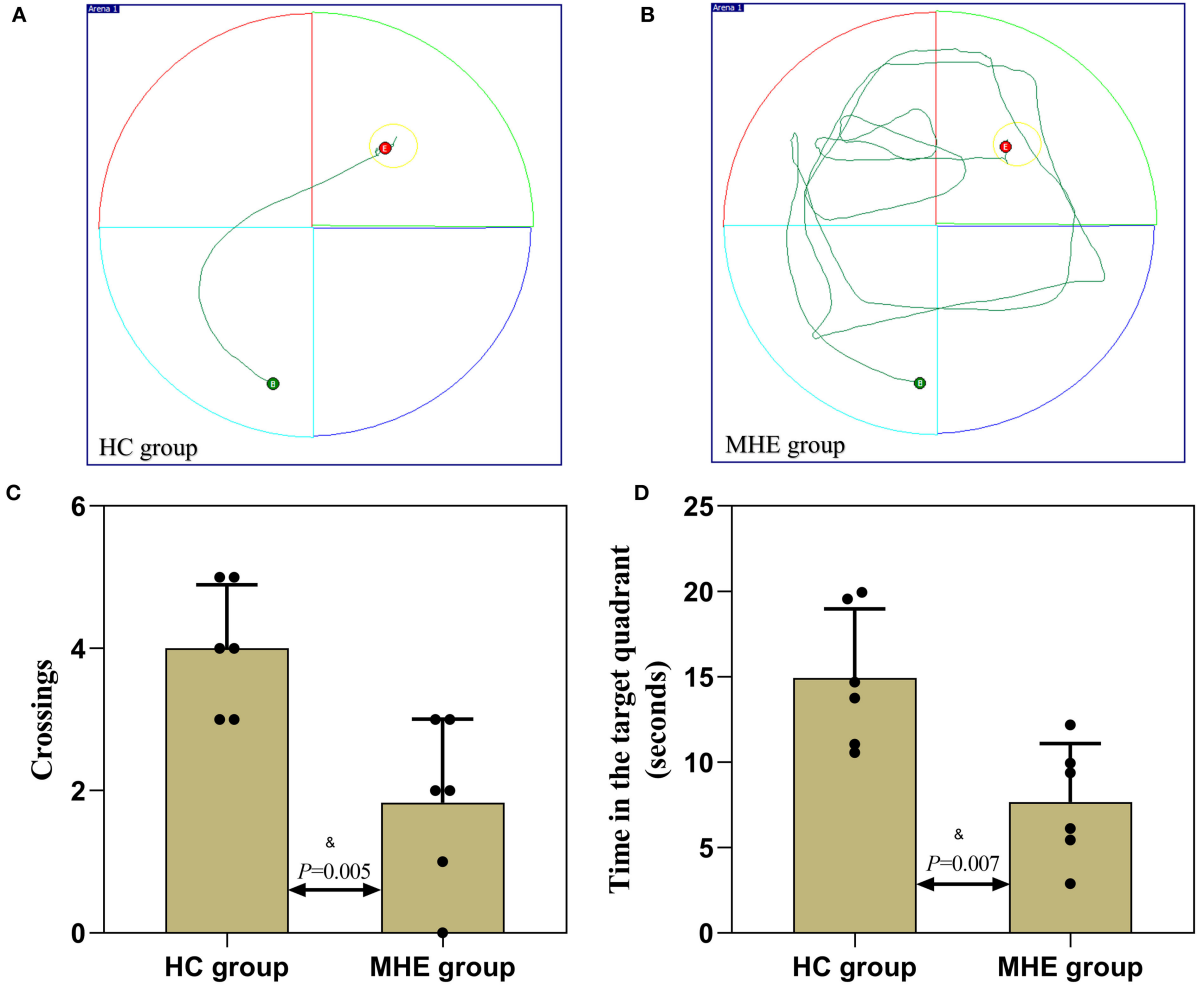
Spatial memory performance of MHE and control rats. **(A,B)** are the representative trace in the water maze task; **(C)** The number of times crossed the platform in the spatial probe test; **(D)** The time spent in the target quadrant in the spatial probe test; ^&^Student's *t*-test.

On the 6th day, the spatial probe test was performed to monitor whether rats remembered the location of the platform. As shown in Figure 4, compared with control rats, MHE rats crossed the platform less often and the time spent in the target quadrant was significantly decreased (*P* < 0.05). The results revealed that the spatial memory and orientation ability of the MHE rats was lower than the control rats.

### Autonomic activity and exploratory behavior assays

**Figure 5:**
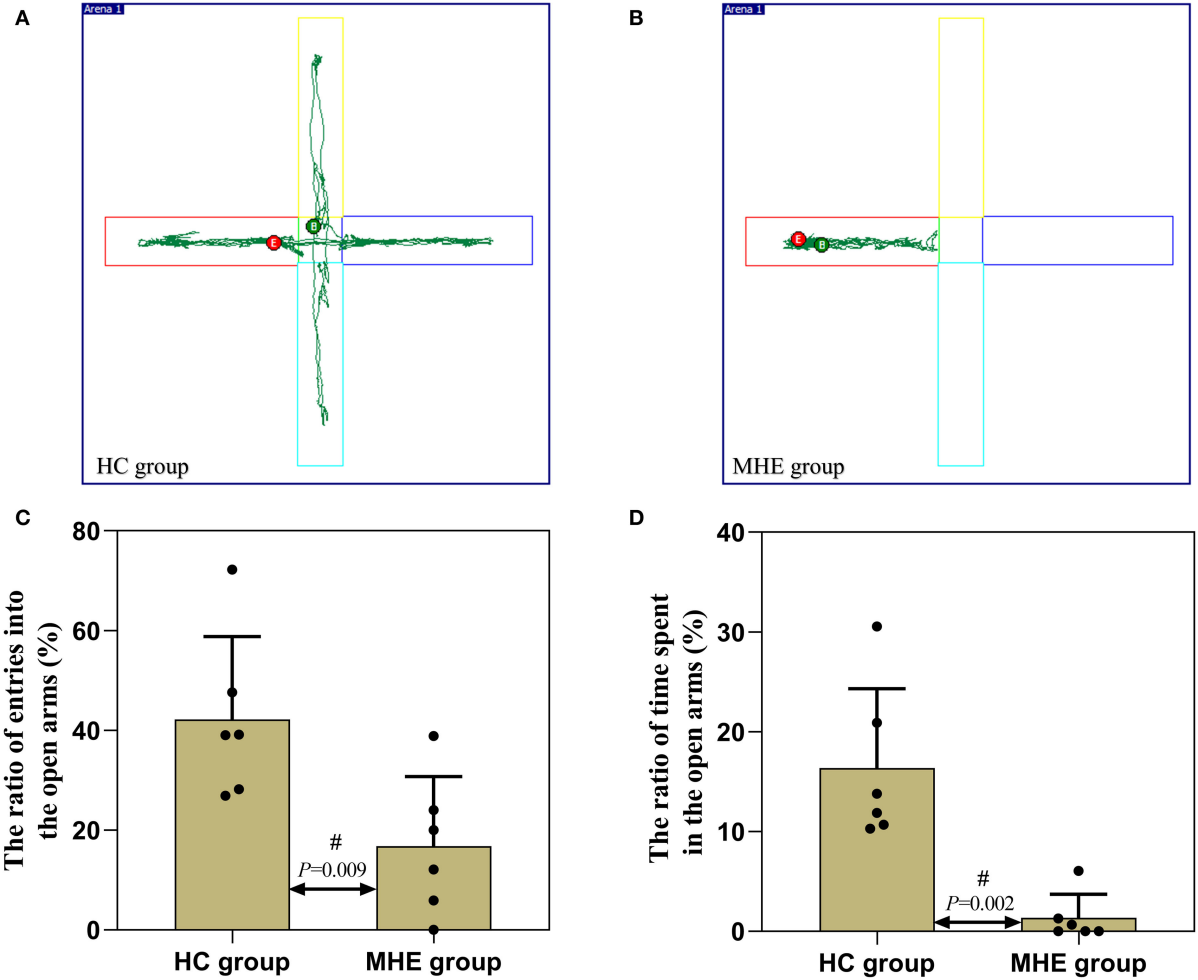
Autonomic activity and exploratory behavior of MHE and control rats. **(A,B)** are the representative trace in the elevated plus maze task; **(C)** The ratio of entries into the open arms; **(D)** The ratio of time spent in the open arms; ^#^Mann-Whitney *U*-test.

In the EPM experiment, we observed that the ratio of entries into the open arms (Figure 5C) and the ratio of time spent in the open arms were decreased than the control rats (*P* < 0.05) (Figure 5D). In comparison with the control rats, MHE rats showed significantly lower levels of autonomous activity and exploratory behavior, a significant impairment of locomotion and exploratory behavior can be seen.

### Changes in serum and tissue BuChE activity of MHE rats

**Figure 6:**
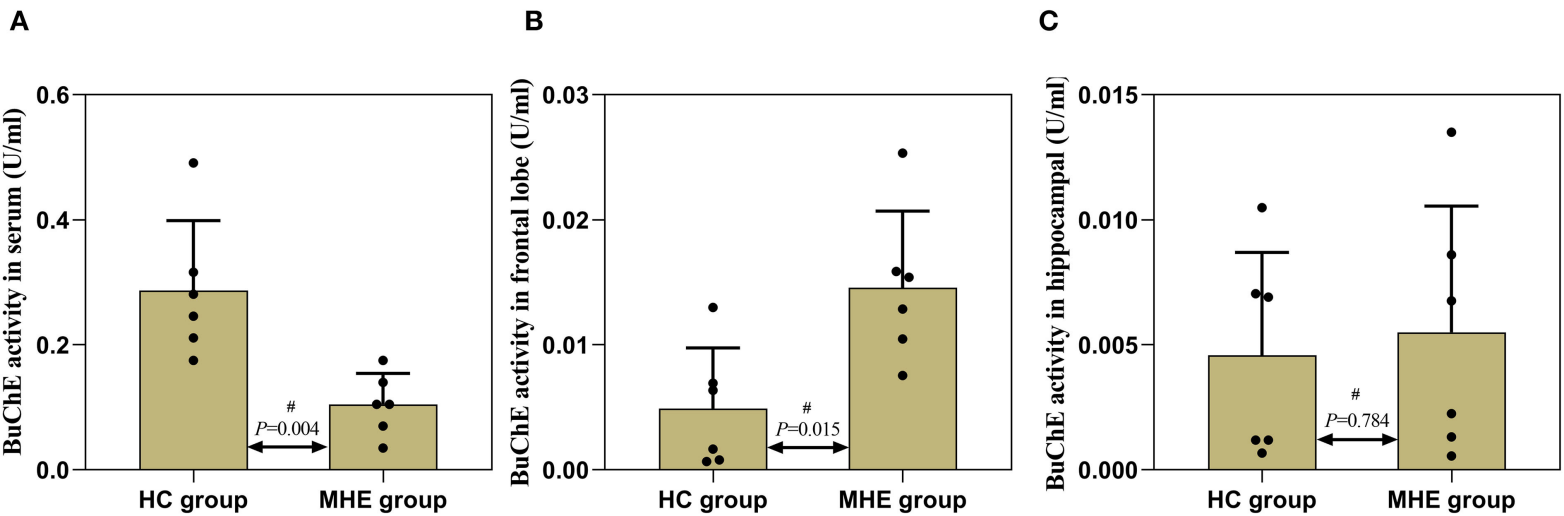
BuChE activity in the peripheral and central of MHE and control rats. **(A)** BuChE activity in serum; **(B)** BuChE activity in frontal lobe tissue; **(C)** BuChE activity in hippocampal tissue; ^#^Mann–Whitney *U*-test.

The detection results of BuChE activity showed that the activity of serum BuChE in the MHE rats was significantly lower than in the control rats (*P* = 0.004) (Figure 6A). BuChE activity was significantly higher in the frontal extract in the MHE rats when compared to the control rats (*P* = 0.015) (Figure 6B), while there was no significant difference in the hippocampal extract (*P* = 0.784) (Figure 6C). The levels of BuChE were normalized by protein contents.

## Discussion

In the present study, we described the changes in peripheral and central BuChE activity in MHE patients and TAA-induced chronic liver failure-related MHE rats. We found that there was a high correlation between the mean ReHo value in the default network of MHE patients and the activity of BuChE. In addition, we observed that there was a strong correlation between BuChE activity and cognitive scale scores. At the same time, we found that peripheral BuChE activity was decreased in MHE patients as well as rats. Interestingly, we also observed a significantly higher BuChE activity in the frontal tissue of MHE rats compared to the control rats. Based on these results, it is tempting to say that changes in peripheral BuChE activity could be a mirror of central BuChE deregulation, which is related to the occurrence of MHE, and cognitive impairment.

BuChE, also known as pseudocholinesterase or non-specific cholinesterase, is a serine hydrolase that can catalyze the hydrolysis of cholinester, to the highest levels in serum and the liver (28, 29). BuChE is expressed in the different neurons of the human brain areas, including the amygdala, hippocampus, and thalamus (30). The unique distribution of BuChE in the central nervous system and its expression in the brain structures involved in cognition has resulted in increasing awareness about its potential role in the regulation of cognitive functions. In the present study, we observed a gradual decrease in BuChE activity during the progression from cirrhosis to MHE. BuChE, as a marker of inflammation and liver injury (31), is affected during liver failure or damage which can lead to a significant decrease in BuChE activity. Meanwhile, Darreh-Shori et al. (32) found that after 12 months of rivastigmine treatment in Alzheimer's disease patients, the percentage reduction of specific activities of serum BuChE was highly correlated with the percentage reduction of BuChE activity in cerebrospinal fluid, which may indirectly indicate that peripheral BuChE activity alterations may affect central BuChE activity. In addition, we also found a significant linear relationship between peripheral BuChE activity with NCT-A and DST scores in MHE patients, while no such correlation was found in the HC group. Based on these results, we believe that the occurrence of cognitive impairment in MHE patients may be related to decreased peripheral BuChE activity. Despite this, the link between brain and serum (BuChE) alterations and behind mechanisms (blood–brain, CSF–brain, and blood–CSF barriers) remains unclear, and it is not clear if those barriers are altered during the disease. But some articles suggest that changes in peripheral biomarkers could affect the central or CSF biomarker deposition and metabolism (33).

Here, one important finding in our study was that the mean ReHo value in the brain region of MHE patients was directly related to the peripheral BuChE. More specifically, we found that BuChE activity negatively correlated with ReHo values in the left middle temporal gyrus and left inferior temporal gyrus, and positively correlated with ReHo value in the right inferior frontal gyrus. The default network is engaged in the maintenance of cognitions related to self-awareness, visual information processing, and visuospatial selective attention (34, 35). The possible correlation between the ReHo within the default network and the BuChE activity in patients with MHE may be explained by the potential role of BuChE in the pathogenesis of MHE, thereby suggesting that the functional impairment of MHE may partly arise from an excess of BuChE in the brain, although this hypothesis requires additional investigation.

To further verify this hypothesis, we used TAA to generate the MHE rat model. Memory, learning, spatial orientation, and exploratory activity were assessed using MWM and EPM. In the MWM experiment, the MHE rats exhibited impairments in spatial memory and orientation, including (1) prolonged latency to reach the platform, (2) decreased number of crossings to the target quadrant, and (3) shorter time spent in the target quadrant (Table 2; Figure 4). These data suggest that MHE rats have deficits in learning and spatial memory compared to the control rats. At the same time, we also found that MHE rats showed lower autonomic activity and exploratory behavior in the EPM test, including the ratio of times entering the open arms and the ratio of time spent in the open arms (Figure 5). In addition, we measured peripheral and central BuChE activity by ELISA in all rats. The results showed that compared with the control rats, the peripheral BuChE activity of the MHE rats was significantly decreased, which was consistent with the results we detected in the MHE patients. Meanwhile, we also found that the activity of BuChE in the frontal lobe extracts of the MHE rats was significantly higher than in the control rats, while no such difference was observed in hippocampal tissue (Figure 6). This may validate our speculation that functional impairment of MHE may partly arise from an excess of BuChE in the brain. Hence, we speculated that once the serum BuChE activity gradually decreases and falls below the corresponding threshold, it might trigger a central response. This in turn can affect the BuChE content in cerebrospinal fluid, and increase the BuChE activity in the brain, which is worth considering. However, Garcia-Ayllon et al., observed no changes in the activity of BuChE in the frontal cortex (12). We speculated that Garcia-Ayllon et al. focused primarily on the acute liver failure model in their study; however, we generated the rat model in the opposite way. It is a chronic process when peripheral BuChE activity decreases, leading to changes in central BuChE activity. This may help explain this difference.

Scholars such as Méndez et al. (36) found that cholinergic neurons are primarily located in areas of the brain associated with cognitive function, and normal cholinergic signaling related to cognitive function is dependent on the action of the neurotransmitter ACh. ACh is the main neurotransmitter synthesized by cholinergic neurons and is hydrolyzed by AChE; in addition to AChE, ACh can also be hydrolyzed by BuChE (37). Therefore, in the present study, the increased activity of BuChE in the frontal lobe of the MHE rats may cause to over-hydrolyze the presynaptic neurotransmitter ACh through an alternative pathway, thereby hindering the transmission of the neurotransmitter, thus resulting in the decrease of extracellular ACh levels, and ultimately affecting information processing and learning acquisition. Due to these, we proposed that the impairment in the ability of MHE rats to learn the spatial orientation and exploration task was dependent on BuChE, which in turn leads to spatial memory deficits and spatial disorientation.

## Limitations

There are some limitations associated with this study. First, the small sample size of this study may be biased and requires further validation by expanding the number of participants in the subsequent studies. Second, we used TAA to generate the MHE rat model; however, this model might not be able to fully mimic the complexity of the human MHE disease. Finally, this study only measured the activity of BuChE in the frontal lobe and hippocampal tissues, and the expression levels of the neurotransmitter ACh were not measured because of the technical limitations of the laboratory. These limitations should be fully considered in future studies to further investigate the role of BuChE in the cognitive function of MHE and its molecular mechanism.

## Conclusion

Overall, our findings showed for the first time that serum BuChE activity was correlated with NCT-A, DST scores, and mean ReHo values in the context of MHE. Furthermore, peripheral BuChE activity was significantly decreased in MHE patients as well as rats, but a significant increase in frontal lobe extracts of MHE rats. These results imply that (1) serum BuChE could facilitate the early diagnosis of MHE, (2) serum BuChE could aid in better characterizing MHE patient profiles during the disease evolution, (3) central BuChE might be deposited as a result of decreased peripheral BuChE activity, and (4) the BuChE may be a potential blood marker for the evolution of MHE disease and is involved in the regulation of cognitive functions.

## Data availability statement

The original contributions presented in the study are included in the article/supplementary material, further inquiries can be directed to the corresponding authors.

## Ethics statement

The animal study was reviewed and approved by Animal Research Committee of Ningxia Medical University. The studies involving human participants were reviewed and approved by General Hospital of Ningxia Medical University. The patients/participants provided their written informed consent to participate in this study. Written informed consent was obtained from the individual(s) for the publication of any potentially identifiable images or data included in this article.

## Author contributions

XW, XD, XY, and PD conceived and designed the study. XY prepared the original draft, reviewed, and edited the final manuscript. XG and KZ revised the manuscript. XH and MW acquired and analyzed the data. WL and WM contributed to data analysis. All authors have read and approved the manuscript.

## Funding

This work was supported by the Natural Science Foundation of Ningxia, China (2022AAC03487), the Science and Technology Key Research Program of Ningxia, China (2019BEG03037), and the Key Laboratory of Craniocerebral Diseases of Ningxia Medical University, China (LNKF202109).

## Conflict of interest

The authors declare that the research was conducted in the absence of any commercial or financial relationships that could be construed as a potential conflict of interest.

## Publisher's note

All claims expressed in this article are solely those of the authors and do not necessarily represent those of their affiliated organizations, or those of the publisher, the editors and the reviewers. Any product that may be evaluated in this article, or claim that may be made by its manufacturer, is not guaranteed or endorsed by the publisher.

## Abbreviations

BuChE: butyrylcholinesterase
MHE: minimal hepatic encephalopathy
HC: healthy control
ReHo: regional homogeneity
NCT-A: number connection test of type A
DST: digital symbol test
ALFF: low-frequency fluctuation
AChE: acetylcholinesterase
ACh: acetylcholine.
